# Comparison of myocardial rotation and torsion in asymptomatic children with single RV, single LV and normal hearts

**DOI:** 10.1186/1532-429X-16-S1-P122

**Published:** 2014-01-16

**Authors:** Ramkumar Krishnamurthy, Cory Noel, Amol Pednekar, Lamya A Atweh, Rajesh Krishnamurthy

**Affiliations:** 1 Radiology, Texas Children's Hospital, Houston, Texas, USA; 2 Pediatric Cardiology, Baylor College of Medicine, Houston, Texas, USA; 3 Philips Healthcare, Houston, Texas, USA

## Background

Abnormal myocardial rotation and torsion have been investigated earlier as markers of ventricular failure1-2. However, there are no previous studies on global or regional torsion in functional single ventricle (SV) or the contribution of torsion to biomechanical differences between single RV (SRV) and single LV (SLV) hearts. Additionally, regional variations in rotation between the ventricular free wall and the septal wall which borders the hypoplastic chamber in SV have not been studied. Purpose: In normal subjects and in asymptomatic patients with SLV and SRV after total cavopulmonary connection (TCPC), to compare: 1) Global rotation (ROTglo). 2) Regional rotation in systole at free wall (ROTfree) and septal wall (ROT sept) for each slice location. 3) Torsion across the ventricle from apex to base.

## Methods

**Figure 1:**
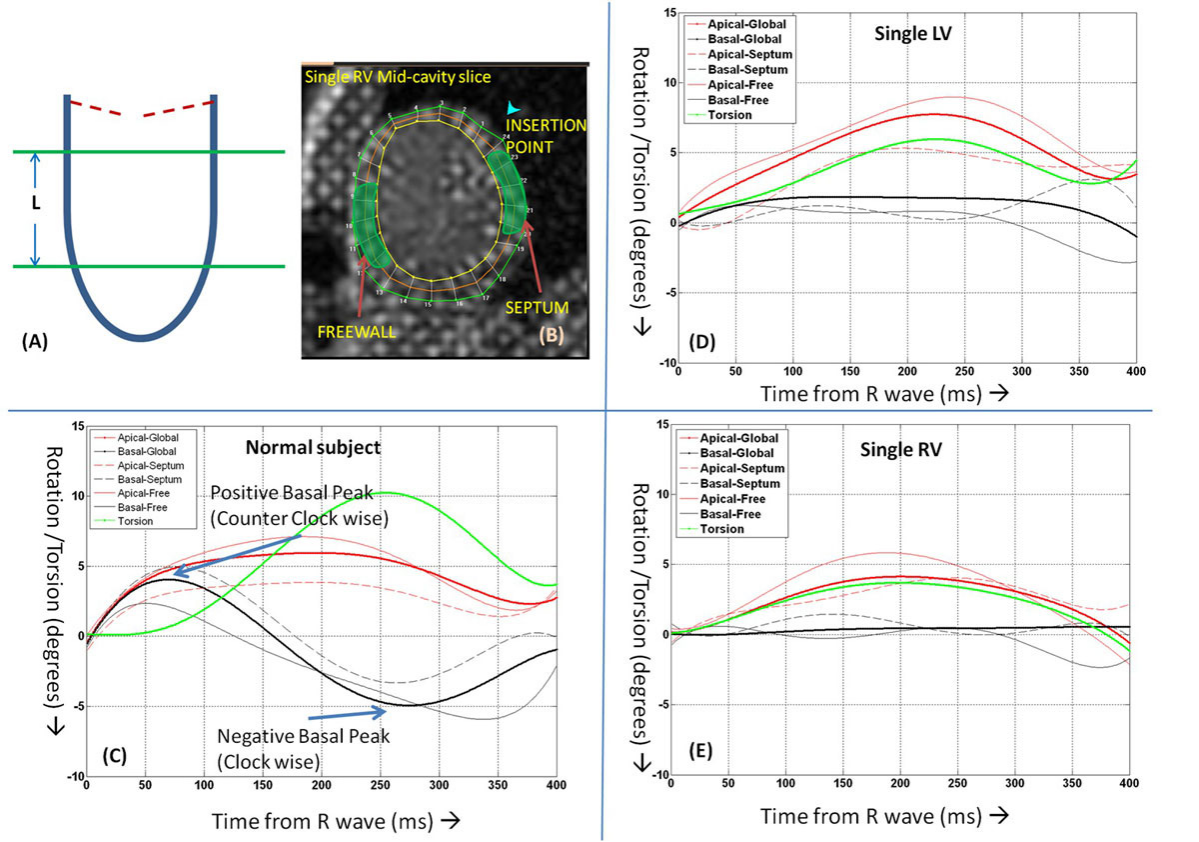
**Schematic (A) showing short axis acquisition of CSPAMM images at the basal and apical levels of the systems ventricle**. A CSPAMM image of a SRV patient (B) at the mid-cavity location is showed with the myocardium segmented, indicating the insertion point, septum and free wall. Representative plots of rotation (global, septal wall and free-wall) is shown for a normal subject (C), patient with systemic single LV (D) and systemic single RV (D). All positive values are counter-clock wise rotation when looked from apex longitudinally. Note the presentce of a substantial positive and negative peak in the basal rotation of a normal subject which are significantly diminished in case of patients with systems single LV and RV (C, D and E), leading to a decreased peak torsion.

We performed a prospective analysis of 18 subjects. Seven subjects had normal cardiac anatomy (age 11.8 +/- 3), six subjects had a SV of right ventricular morphology (age: 11.4 +/- 2.3), and 5 had a SV of left ventricular morphology (age 12.7 +/- 4.2). Acquisition Protocol: On a 1.5T Philips Acheiva MRI scanner, one long-axis (4-chamber), two short axis (SAX) slices at basal and apical locations were obtained in all subjects using Complementary Spatial Modulation of Magnetization (CSPAMM)3 technique with a temporal resolution of 35 to 50 ms, spatial resolution 2*2*6 mm. Data Analysis: Rotation (in degrees) across all cardiac phases within 400 ms of the R wave (to account for tag fading) was calculated for both the slices using DiagnosoftTM. ROTglo, ROTsept, and ROTfree were then calculated for each patient by performing a polynomial fit. Global Torsion (τ) was calculated as difference in global rotation between apical and basal slices. Normalized global torsion (τNorm) was calculated as τ/L, where L is the distance between the apical and basal slice (Figure 1A). Counter clockwise rotation (when viewed from apex) was considered positive.

## Results

**Figure 2:**
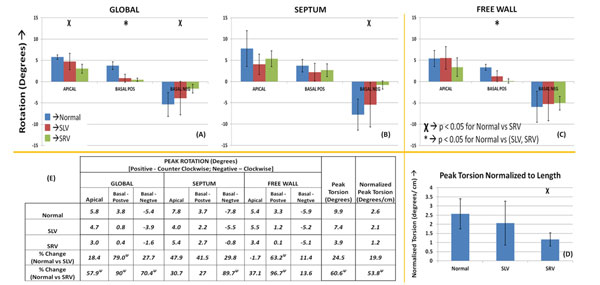
**Bar plots showing the peak rotation of short axis slices in normal subjects and in patients with systemic single ventricles**. It was seen that the basal rotation was significantly reduced in both SLV and SRV subjects (A, B and C). In the case of SRV, the global apical rotation (A) and septal basal negative rotation were significantly reduced (B). The peak torsion (not shown) and peak torsion normalized to length between apex and basal slice (D) were reduced in both SLV and SRV wrt. normal subjects. This reduction was statistically significant for SRV (see table (E)). Please refer to bar plots for standard deviation values. Ψ→p < 0.05 for Student's t-test.

The following observations were made (See Figure 2 for all results) in SLV and SRV patients with respect to (wrt) normal data: 1. Significant reduction in global basal rotation (counter clockwise) in both SLV (79%) and SRV (90%) patients. 2. Decreased peak τ and τNorm in SLV and SRV (p < 0.05). 3. Basal rotation more severely impaired than apical rotation in SRV and SLV patients. 4. SRV patients had more severely impaired rotation than SLV (significantly reduced global and septal clock-wise basal rotation).

## Conclusions

SLV and SRV patient demonstrated a significantly impaired rotation compared to normal subjects, with SRV patients more severely affected. In single ventricular patients, basal rotation is more impaired compared to apical rotation, suggesting a deleterious effect of the hypoplastic chamber connected to the base.

## Funding

None.
